# Medium supplementation can influence the human ovarian cells in vitro

**DOI:** 10.1186/s13048-022-01081-2

**Published:** 2022-12-26

**Authors:** Arezoo Dadashzadeh, Saeid Moghassemi, Monika Grubliauskaité, Hanne Vlieghe, Davide Brusa, Christiani A. Amorim

**Affiliations:** 1 Pôle de Recherche en Physiopathologie de la Reproduction, Institut de Recherche Expérimentale et Clinique, Université Catholique de Louvain, Avenue Hippocrate 55, bte B1.55.03, 1200 Brussels, Belgium; 2 Department of Biobank, National Cancer Institute, 08660 Vilnius, Lithuania; 3 CytoFlux-Flow Cytometry and Cell Sorting Platform, Institut de Recherche Expérimentale et Clinique, Université Catholique de Louvain, 1200 Brussels, Belgium

**Keywords:** Cell characterization, Ovary, Fetal bovine serum, Human serum albumin, Platelet lysate, KnockOut serum

## Abstract

**Background:**

Cells are an essential part of the triple principles of tissue engineering and a crucial component of the engineered ovary as they can induce angiogenesis, synthesize extracellular matrix and influence follicle development. Here, we hypothesize that by changing the medium supplementation, we can obtain different cell populations isolated from the human ovary to use in the engineered ovary. To this end, we have in vitro cultured cells isolated from the menopausal ovarian cortex using different additives: KnockOut serum replacement (KO), fetal bovine serum (FBS), human serum albumin (HSA), and platelet lysate (PL).

**Results:**

Our results showed that most cells soon after isolation (pre-culture, control) and cells in KO and FBS groups were CD31- CD34- (D0: vs. CD31-CD34+, CD31 + CD34+, and CD31 + CD34- *p* < 0.0001; KO: vs. CD31-CD34+, CD31 + CD34+, and CD31 + CD34- *p* < 0.0001; FBS: vs. CD31-CD34+ and CD31 + CD34+ *p* < 0.001, and vs. CD31 + CD34- *p* < 0.01). Moreover, a deeper analysis of the CD31-CD34- population demonstrated a significant augmentation (more than 86%) of the CD73+ and CD90+ cells (possibly fibroblasts, mesenchymal stem cells, or pericytes) in KO- and FBS-based media compared to the control (around 16%; *p* < 0.001). Still, in the CD31-CD34- population, we found a higher proportion (60%) of CD90+ and PDPN+ cells (fibroblast-like cells) compared to the control (around 7%; vs PL and KO *p* < 0.01 and vs FBS *p* < 0.001). Additionally, around 70% of cells in KO- and FBS-based media were positive for CD105 and CD146, which may indicate an increase in the number of pericytes in these media compared to a low percentage (4%) in the control group (vs KO and FBS *p* < 0.001). On the other hand, we remarked a significant decrease of CD31- CD34+ cells after in vitro culture using all different medium additives (HSA vs D0 *p* < 0.001, PL, KO, and FBS vs D0 *P* < 0.01). We also observed a significant increase in epithelial cells (CD326+) when the medium was supplemented with KO (vs D0 *p* < 0.05). Interestingly, HSA and PL showed more lymphatic endothelial cells compared to other groups (CD31 + CD34+: HSA and PL vs KO and FBS *p* < 0.05; CD31 + CD34 + CD90 + PDPN+: HSA and PL vs D0 *p* < 0.01).

**Conclusion:**

Our results demonstrate that medium additives can influence the cell populations, which serve as building blocks for the engineered tissue. Therefore, according to the final application, different media can be used in vitro to favor different cell types, which will be incorporated into a functional matrix.

## Background

The ovaries are female reproductive organs that contain four layers, including the germinal epithelium layer, the collagenous connective tissue, the cortex holding preantral follicles, and the medulla comprising loose connective tissue, blood vessels, and antral follicles. Follicles are the main components of the ovary containing oocytes and granulosa cells [1]. Females are born with a limited number of oocytes (around 1–2 million), which are placed in primordial follicles [2]. Menopause begins when the woman’s follicular reserve is depleted, and she is no longer able to conceive naturally [3]. On the other hand, studies reported that both pre- and post-menopausal ovaries contain pluripotent/multipotent stem cells that may differentiate into multiple cell lineages [4–6]. Stimpfel et al. [4] investigated the adipogenic, osteogenic, neural, and pancreatic differentiation ability of stem cells derived from pre- and post-menopausal ovaries and demonstrated the high plasticity of the stem cells isolated from these ovaries. Somatic stem cells are a subgroup of normal tissues with self-renewal ability and the potential to create lineage-committed daughter cells, which are important for tissue regeneration and repair [7]. On the other hand, the culture of stem cells in different media (for instance, supplemented media with fetal calf serum, human serum, or platelet lysate) could affect their proliferation rate, function, and phenotype [8].

Fan et al. [9] and Wagner et al. [10] provided a map of isolated cells from reproductive-age ovaries and identified six groups of cells, including endothelial, immune, granulosa, smooth muscle, theca, and stroma cells. In tissue engineering, cells play a critical role in the constructed tissue. For instance, endothelial cells could accelerate vascularization, stem cells could improve regeneration, or theca cells are necessary for follicle development [1, 11–13]. Therefore, it is essential to identify various cell types in a tissue and their fate after culturing in different media to optimize the design of an engineered tissue. In this study, we characterize isolated cells from menopausal ovarian cortex before and after in vitro culture using four different supplements: fetal bovine serum (FBS), human serum albumin (HSA), platelet lysate (PL), and KnockOut serum replacement (KO).

## Methods

### Ovarian stromal cell isolation

The ovarian tissue biopsies were collected from post-menopausal women (51–68 years old) and transferred to the laboratory in Dulbecco’s phosphate-buffered saline (DPBS; 14,190,144; Gibco). The ovarian cortical fragments were frozen using our standard method after the medullar section was removed [14]. After thawing of the ovarian samples, stromal cells were isolated following our published protocols [12, 15–17]. Briefly, a tissue chopper (Mickel Laboratory, the UK) set to 0.5 mm was utilized for the mechanical mincing of tissues, and then the enzymatic digestion of chopped tissues was performed by Liberase DH (05401054001; Sigma-Aldrich, Germany) and DNAse I (10,104,159,001; Sigma-Aldrich) at 37 °C for 75 minutes. Then, DPBS supplemented with 10% heat-inactivated fetal bovine serum (FBS; 16,140–071; Gibco, Belgium) was added to the digested tissue suspension to inactivate enzymatic reactions. The suspension was then centrifuged (500 g, 10 min) after filtering through 80 (NY8002500; Millipore, Belgium) and 30 μm (NY3002500; Millipore) nylon net filters. After cell counting, the cells were divided into 5 groups: cells for freezing (D0) and in vitro culture using FBS, HSA (Cliniques Universitaires Saint Luc, Belgium), PL (SER-HPL-GROPRO, Zenbio, USA), and KO (10,828,028; Gibco, Belgium).

### Cell culture

Each cell culture medium consisted of Dulbecco’s modified Eagle’s medium F-12 nutrient mixture (DMEM/F12; 21,041–025; Gibco), 1% antibiotic and antimycotic (Anti-Anti; A5955; Gibco), and 10% FBS, HSA, PL, or KO. The cells were in vitro cultured at 37 °C in a humidified incubator with 5% CO_2_ and the medium was replaced every other day. After 16 days, the cells were detached using Accutase (A6964; Sigma-Aldrich) to perform multiparametric flow cytometry analysis. Furthermore, the light microscopy analysis on cell morphology and proliferation on days 5, 7, 9, and 15 was performed.

### Flow cytometry

Human ovarian isolated and cultured cells were processed for flow cytometry according to the Feisst et al. [18] procedure to determine the cell populations. Before staining, the frozen-thawed cells were washed and incubated in FBS culture medium at 37 °C, 5% CO_2_ for 1 h. After washing with DPBS, frozen-thawed and cultured cells were stained with the following antibodies (Table 1): CD326 (324,233; BioLegend, the Netherlands), CD34 (343,615; BioLegend), CD31 (303,131; BioLegend), CD90 (328,113; BioLegend), CD73 (344,007; BioLegend), podoplanin (PDPN, 337005; BioLegend), CD146 (361,021; BioLegend), and CD105 (323,205; BioLegend) and dark-incubated at room temperature for 10 min.

**Table 1:** CD markers used in cell sorting

CD markers	Cell types
CD326	Epithelial cells
CD34	Hematopoietic stem cells, endothelial progenitor cells
CD31	Endothelial cells
CD90	Stem cells, mesenchymal stem cells, fibroblasts
PDPN	Fibroblast, cancer-associated fibroblasts (CAFs)
CD73	Multiple cells, immunosuppressive enzyme
CD105	Endothelial cells, neoangiogenesis
CD146	Mesenchymal stem cells, angioblasts, endothelial cells, periendothelial cells

At least 10,000 events were analyzed by BD FACSCanto™ II Clinical Flow Cytometry System (BD Biosciences, Belgium). FlowJo software (BD Biosciences, USA) was used for data analysis.

### Statistical analysis

One-way ANOVA was used to examine the data and statistical significance was determined by *P* values less than 0.05. The quantitative data were presented as mean ± SD and a single sample standard deviation is represented by the error bars in graphs.

## Results and discussion

### Flow cytometry

In order to investigate the identity of ovarian stromal cells after isolation (D0) and culture in different media supplemented by HSA, FBS, PL, and KO, the cells were stained with 8 cell markers. These supplements are different from each other and their composition can therefore trigger different cell behaviors. FBS is an undefined serum, and its composition varies from lot to lot and depends on the diet and environment of a pregnant female. Indeed, Zheng et al. [19] investigated the growth of adult retinal pigment epithelial cells in three different FBS batches and reported that the growth rate of cells was significantly higher in one of the batches compared to others. While it is not possible to establish the exact composition of FBS, some of its contents have already been described in the literature, such as different types of hormones (follicle-stimulating hormone, glucagon, insulin, and thyroid hormones), growth factors and cytokines (basic fibroblast growth factor, endothelial cell growth factor, epidermal growth factor, and fibroblast growth factor) and other proteins (albumin, fibronectin, laminin, and transferrin) [20]. To reduce uncontrolled cell differentiation and avoid endotoxin and large variability observed in the FBS, this supplement has been replaced by other additives, such as serum-free medium, animal-free components, such as PL, and more defined serum replacement such as KO [21–23].

Studies have shown that undesired differentiation of cells cultured in a medium supplemented with KO is lower than FBS [24, 25]. KO is composed of well-defined growth factors, amino acids, vitamins, antioxidants, trace elements, and proteins, including transferrin (iron-saturated), insulin, and lipid-rich albumin (AlbuMAX) [26]. Nevertheless, it is important to bear in mind that the exact composition of KO is not provided by its supplier. Moreover, KO is not the completely free of animal components because of the presence of AlbuMAX [27] and embryo extracts [28]. Albumin is an important protein with antioxidant function as well as a role in binding and carrying biological important factors for cells. Moreover, surfaces coated with albumin cause non-adhesive surfaces, preventing cell attachment [29, 30]. Although HSA has been employed in cell culture, such as human endothelial cells, in which HSA acted as an apoptotic inhibitor [31], studies from human islet and embryo culture in FBS or FBS + HSA indicated superior results than HSA alone regarding oxygen consumption rate per DNA content or blastocyst implantation rate [32, 33]. A more recent alternative to FBS is PL, an animal-free product, produced from human platelets, which are lysed several freezing and thawing cycles. PL contains HSA as a major protein component as well as different types of growth factors, such as fibroblast growth factor (FGF), endothelial growth factor (EGF), platelet-derived growth factors (PDGF), vascular endothelial growth factor (VEGF), transforming growth factor (TGF), insulin-like growth factor-1 (IGF-1), brain-derived neurotrophic factor (BDNF), and epidermal growth factor (EGF) originated from human plasma and platelet components [34, 35]. However, PL is also a donor-related product, and a decrease in the concentration of PDGF, TGF, FGF, or IGF with an increase in donor age has been shown [36]. Therefore, in order to decrease batch variation, PL is generally produced from multiple donors. Based on such differences in these supplement (HSA, FBS, PL, and KO) compositions, it is indeed expected that they can regulate cell behavior differently. For instance, mesenchymal stem cells proliferate faster in medium supplemented with PL than with FBS [37].

**Fig. 1:**
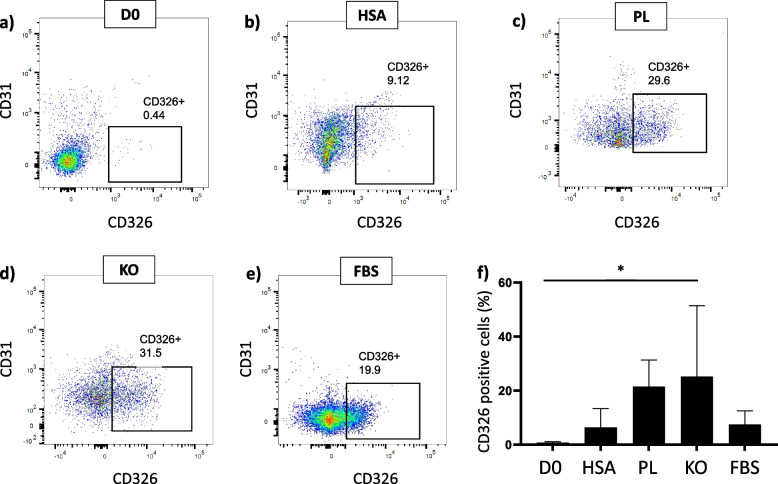
Epithelial cell distribution in different samples. Representative dot plot from cells at D0 (**a**), or in vitro cultured in HSA (**b**), PL (**c**), KO (**d**), and FBS (**e**). CD326+ epithelial cells of samples in percent (**f**); *n* ≥ 3, mean ± SD, *p < 0.05

In vitro culture in medium supplemented with KO medium yielded a significantly higher proportion of CD326+ cells (epithelial-like cells) compared to D0 (25.19 ± 22.7% vs. 0.72 ± 0.38%; *p* < 0.05) (Fig. 1). On the other hand, other medium additives had no significant differences in the CD326+ population.

**Fig. 2:**
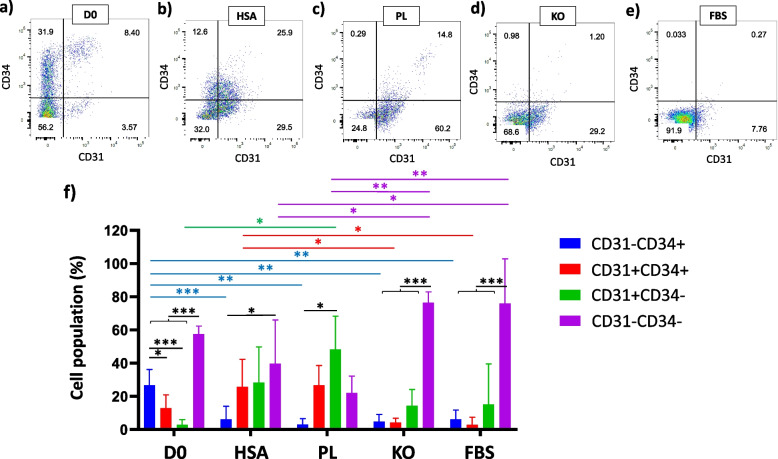
Representative dot plots from the investigation of CD31 and CD34 stained cells in D0 (**a**), or cultured in HSA (**b**), PL (**c**), KO (**d**), and FBS (**e**). A graph represents the mean ± SD of main gates (CD31-CD34+, CD31 + CD34+, CD31 + CD34-, and CD31-CD34-) (**f**); black asterisks represent significant differences between main gates of each sample; blue, red, green, and purple asterisks represent significant differences between CD31-CD34+, CD31 + CD34+, CD31 + CD34-, and CD31-CD34- gates of all samples, respectively; *n* ≥ 3, mean ± SD; *p < 0.05; **p < 0.01; ***p < 0.001

CD326- cells were further analyzed for the CD31 and CD34 (CD31-CD34+, CD31 + CD34+, CD31 + CD34-, and CD31-CD34-). Although CD31-CD34- cells in PL samples had no significant difference from other groups, other samples, except PL, indicated significant differences between CD31-CD34- and CD31-CD34+, CD31 + CD34+, and CD31 + CD34- (D0: vs. CD31-CD34+, CD31 + CD34+, and CD31 + CD34- *p* < 0.0001; KO: vs. CD31-CD34+, CD31 + CD34+, and CD31 + CD34- *p* < 0.0001; FBS: vs. CD31-CD34+ and CD31 + CD34+ *p* < 0.001, and vs. CD31 + CD34- *p* < 0.01). For HSA, CD31-CD34- had only a significant difference with CD31-CD34+ (*p* < 0.05) (Fig. 2).

**Fig. 3:**
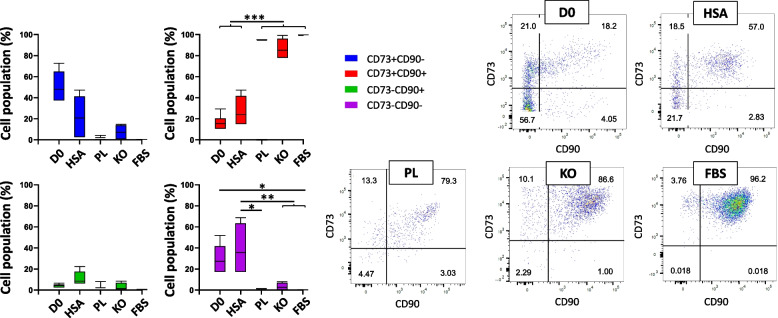
Representative dot plot for identification of cells in CD31-CD34- gates of samples. Evaluation of expressing CD90 and CD73 of CD31-CD34- cells in D0, HSA, PL, KO, and FBS samples; *n* ≥ 3, mean ± SD; *p < 0.05; **p < 0.01; ***p < 0.001

**Fig. 4:**
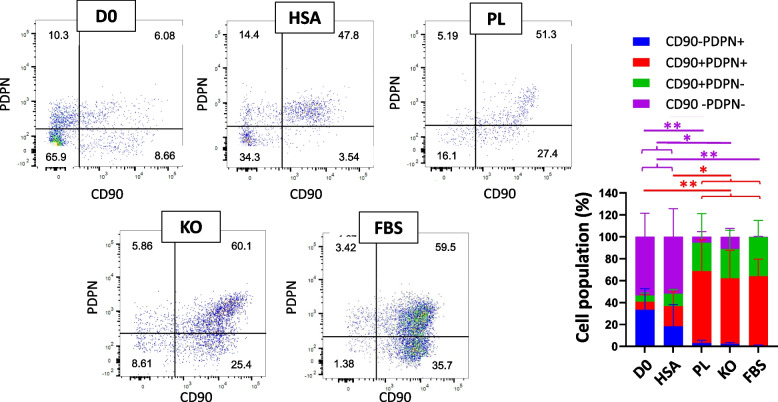
Representative dot plot for identification of cells in CD31-CD34- gates of samples. Evaluation of expressing CD90 and PDPN of CD31-CD34- cells in D0, HSA, PL, KO, and FBS samples; *n* ≥ 3, mean ± SD; *p < 0.05; **p < 0.01; ***p < 0.001

**Fig. 5:**
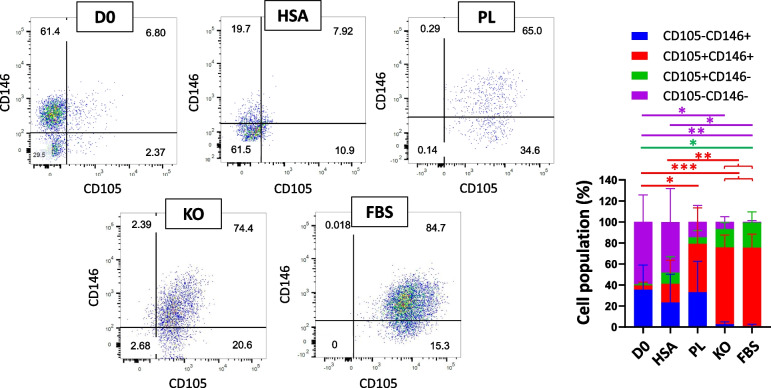
Representative dot plot for identification of cells in CD31-CD34- gates of samples. Evaluation of expressing CD105 and CD146 of CD31-CD34- cells in D0, HSA, PL, KO, and FBS samples; *n* ≥ 3, mean ± SD; *p < 0.05; **p < 0.01; ***p < 0.001

The CD31-CD34- cells could be fibroblast or mesenchymal stem cells (MSCs). For further investigations, the expression of CD73, CD90, PDPN, CD146 and CD105 was assessed (Figs. 3, 4, and 5). Each sample had more than 5% cells in main gates (CD31-CD34+, CD31 + CD34+, CD31 + CD34-, and CD31-CD34-) considered for this analysis. One of the criteria for identifying MSCs is CD34-, CD73+, CD90+, and CD105+ [38]. Although fibroblasts express CD73, CD90, and CD105, as well [39], the PDPN could be another marker for stromal fibroblasts to be distinguished from MSCs [40, 41]. In addition, endothelial cells could be determined by the expression of CD31 and the perivascular marker CD146 [42].

Looking at CD31-CD34- cells, CD90 and CD73 gates (Fig. 3) reveals that the cells after culturing in different media significantly increased in CD90+ and CD73+ quantities (in HSA, PL, KO, and FBS were 35.38 ± 21.23%, 92.6 ± 3.19%, 86.45 ± 8.26%, and 99.40 ± 0.24% respectively) compared to D0 (16.31 ± 6.51%, vs. HSA, PL, KO, and FBS; *p* < 0.001). Regarding the majority of cell population in KO and FBS samples are in CD31-CD34- gate and, also, around 86% and 99% cells of CD31-CD34- gate in KO and FBS are CD90+ and CD73+, respectively, major cell types in KO and FBS supplemented samples could be characterized as MSCs, fibroblast, or pericytes, which are CD34-, CD73+, and CD90 + .

Furthermore, cell populations of CD31-CD34-CD90 + PDPN+ rose after culturing in PL (65.77 ± 23.36%; vs. D0; *p* < 0.01), KO (59.95 ± 22.18%; vs. D0; *p* < 0.01), and FBS (63.45 ± 13.39%; vs. D0; *p* < 0.001) (Fig. 4). The results show that around 60% of CD31-CD34- in PL, KO, and FBS could have fibroblast-like phenotype cells, which was around 7% on D0. The cells cultured in HSA had no significant difference with D0 in the number of CD31-CD34- gates cells expressing CD90 and PDPN. Besides, FBS, KO, and PL demonstrated decreasing cell percentage in CD31-CD34- identified CD90-CD73-, CD90-PDPN-, and CD105-CD146- compared to HSA and D0 samples (Figs. 3, 4, and 5).

A surge was also observed in the CD31-CD34- CD105 + CD146+ after culturing cells in different media from 4.13 ± 2.86% on D0 to 46.07 ± 28.03% in PL (vs. D0; *p* < 0.05), 73.23 ± 10.16% in KO (vs. D0; *p* < 0.001), and 74.68 ± 11.14% in FBS (vs. D0; *p* < 0.001) (Fig. 5). On the other hand, HSA had no significant difference in cell population characterized CD31-CD34-CD105+ CD146+ compared to D0.

While pericytes and MSCs express CD44, CD90, CD73, and CD105 [43] markers and pericytes and bone marrow-derived MSCs are CD146+ and CD34- [44, 45], as regards the cells in this experiment isolated from the ovarian cortex, not bone tissue, the CD31-CD34-CD105 + CD146+ could indicate pericytes (perivascular cells). These are fibroblast-like cells and around endothelial cells found in the endothelium basement [46, 47]. Therefore, the results could demonstrate that although a small number of pericytes characterized D0 samples, they could proliferate and increase their number, especially in KO- and FBS-supplemented media. On the other hand, cells in D0 and HSA were characterized by a higher number of cells in CD31-CD34-CD105-CD146- compared to KO and FBS (D0 vs. KO; *p* < 0.05, vs. FBS; *p* < 0.01, HSA vs. FBS; *p* < 0.05) (Fig. 5).

As CD34 is a marker of progenitor cells [48–51] and endothelial cells [52], CD31-CD34+ could be progenitor cells and CD31 + CD34+ could represent endothelial progenitor cells. Although cells cultured in different media showed no significant differences between cell populations in CD31-CD34+ and CD31 + CD34+ gates, these two gates on D0 had a significant difference in cell populations (*p* < 0.05; 26.75 ± 8.6% and 12.87 ± 7.34%, respectively) (Fig. 2). This may indicate differentiation of cells in CD31-CD34+ gate to other cell types when cells cultured in different media, which the average cell population in CD31-CD34+ gate decrease to 6.07 ± 7.34% in HSA (vs. D0; *p* < 0.001), 2.99 ± 2.92% in PL (vs. D0; *p* < 0.01), 4.85 ± 3.63% in KO (vs. D0; *p* < 0.01), and 6.1 ± 4.84% in FBS (vs. D0; *p* < 0.01) (Fig. 2). Moreover, HSA compared to KO and FBS in CD31 + CD34+ gate (*p* < 0.05) and PL compared to D0 in CD31 + CD34- gate (*p* < 0.05) had a higher cell number (Fig. 2), demonstrating differentiation of cells to the endothelial cells (CD31 + CD34-) in PL or endothelial progenitor cells (CD31 + CD34+) in HSA-based media.

**Fig. 6:**
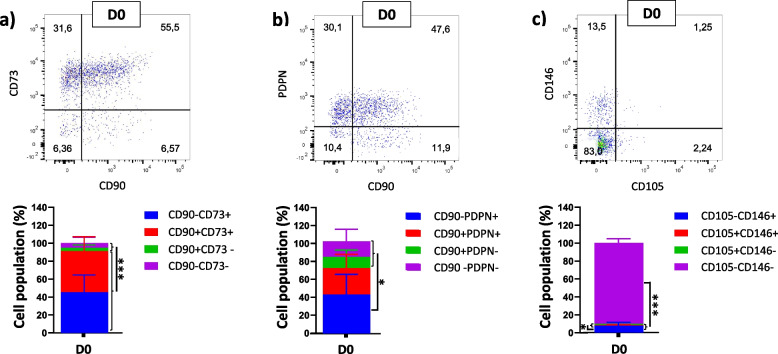
Representative dot plot for characterizing CD31-CD34+ gate of D0 with different antibodies: CD90 and CD73 (**a**), CD90 and PDPN (**b**), and CD105 and CD146 (**c**); *n* ≥ 3, mean ± SD; *p < 0.05; ***p < 0.001

The further evaluation of the cell population in CD31-CD34+ gates stained by extra antibodies (CD90 and CD73, CD90 and PDPN, and CD105 and CD146) reveals that considering the expression of CD90 and CD73 on D0, the majority of cells could be divided into two groups (Fig. 6): CD31-CD34 + CD90-CD73+ and CD31-CD34 + CD90 + CD73+ (45.33 ± 17.5% and 45.82 ± 14.82%, respectively). The further analysis of the CD31-CD34+ gate was not considered for HSA, PL, KO, and FBS, which had cell populations of less than 5%. As hematopoietic stem cells express CD34 and CD90 [53], around 45% of cells in the CD31-CD34+ gate could be identified as hematopoietic stem cells in D0 samples. Moreover, considering CD31-CD34 + CD90PDPN and CD31-CD34 + CD105CD146 gates on D0, the majority of cell populations belonged to CD31-CD34 + CD90-PDPN+ (42.55 ± 19.87%) and CD31-CD34 + CD105-CD146- (90.33 ± 4.22%) (Fig. 6). Ho et al. [54] demonstrated that maturate fibroblasts has a profile of CD34 + CD90-, so we can consider the CD31-CD34 + CD90-PDPN+ cells as maturate fibroblasts existing in the ovarian cortex.

**Fig. 7:**
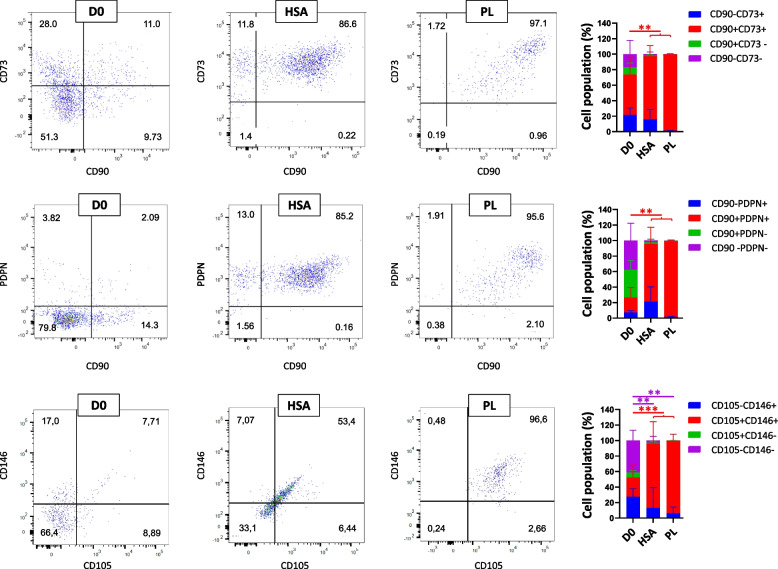
Representative dot plot for discovering CD90CD73, CD90PDPN, and CD105CD146 expressions of cells in CD31 + CD34+ gate of D0, HSA, and PL samples; *n* ≥ 3, mean ± SD; **p < 0.01; ***p < 0.001

Since CD31 + CD34+ gate of FBS and KO had a cell population of less than 5%, this gate was not considered for more evaluation in these media. Cells in culture in HSA- and PL-based media indicated increasing in CD31 + CD34 + CD90 + CD73+ cell populations (81.33 ± 12.57% and 98.12 ± 0.75%, respectively) compared to D0 (52.15 ± 20.52%; vs. HSA and vs. PL; *p* < 0.01) (Fig. 7). Moreover, when HSA or PL was used as media additives, most cell phenotypes changed from 35.75 ± 11.12% CD31 + CD34 + CD90 + PDPN- and 37.87 ± 20.17% CD31 + CD34 + CD90-PDPN- in D0 to 74.33 ± 19.59% and 97.4 ± 1.27% CD31 + CD34 + CD90 + PDPN+ in HSA and PL, respectively. Therefore, the main population of cells in CD31 + CD34+ changed their phenotype from PDPN- on D0 to the PDPN+ cells when cultured in HSA and PL. Considering that lymphatic endothelial cells are CD31 + CD34 + CD90 + PDPN+ [55, 56], around 74 and 97% of cells in the CD31 + CD34+ gate may be recognized as lymphatic endothelial cells in HSA and PL, respectively (vs D0 *p* < 0.01).

Furthermore, the phenotype change was observed in CD31 + CD34 + CD105CD146, which after culturing cells in media containing HSA and PL, the cell populations in CD31 + CD34+ CD105 + CD146+ reached 83.17 ± 25.65% and 93.6 ± 6.85% in HSA and PL, respectively, from 24.52 ± 14.56% in D0 (vs. HSA and PL; *p* < 0.001). On the other hand, CD31 + CD34+ CD105-CD146- cell populations were significantly decreased in HSA and PL (2.8 ± 4.77%; vs. D0; *p* < 0.01 and 0.10 ± 0.14% vs. D0; *p* < 0.05, respectively) in comparison to D0 (41.33 ± 12.17%). This surge of CD31 + CD34+ CD105+ CD146+ cells in PL and HSA showed the ability of these additives to increase the number of endothelial cells expressing CD31, CD34, CD105, and CD146.

**Fig. 8:**
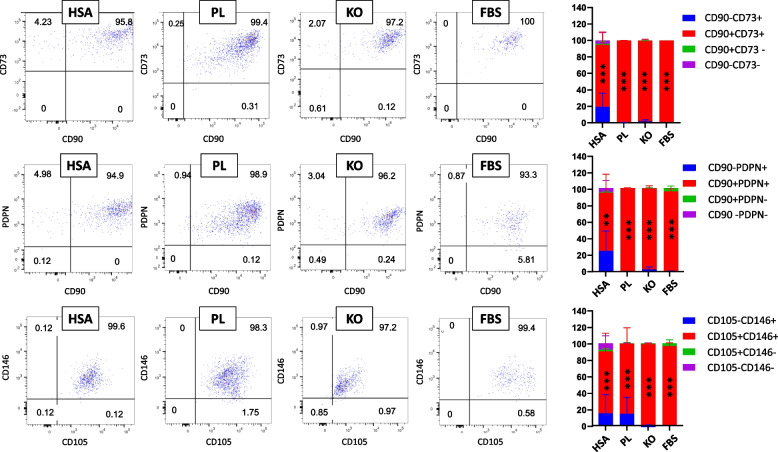
Representative dot plot for CD90, CD73, CD105, and CD146 expressed cells of CD31 + CD34- gate in different media (HSA, PL, KO, and FBS); *n* ≥ 3, mean ± SD; the asterisks demonstrate significant differences of CD90 + CD73+, CD90 + PDPN+, and CD105 + CD146+ with other gates; **p < 0.01; ***p < 0.001

CD31 + CD34- gates of samples, except D0, were also checked for other antibodies (Fig. 8). The results showed that the majority of cells in all culture conditions characterized as CD31 + CD34-CD90 + CD73+ (in HSA, PL, KO, and FBS were 75.28 ± 14.48%, 99.53 ± 0.19%, 92.53 ± 9.28%, and 96.58 ± 5.93%, respectively), CD31 + CD34-CD90 + PDPN+ (in HSA, PL, KO, and FBS were 69.27 ± 20.52, 99.27 ± 0.26%, 91.55 ± 8.94%, and 92.73 ± 7.47%, respectively), and CD31 + CD34-CD105 + CD146+ (in HSA, PL, KO, and FBS were 75.07 ± 20.24%, 85.13 ± 15.74%, 96.73 ± 3.13%, and 93.73 ± 6.98%, respectively) (Fig. 8). These results demonstrate that the most cell population of CD31 + CD34- in the different media could be endothelial cells, which may be positive for CD31, CD90, CD73, CD105, and CD146. However, blood vessel endothelial cells are PDPN- and lymphatic endothelial cells are PDPN+ [55]. Since more than 90% of cells in the CD31 + CD34- gate of PL, KO, and FBS are CD90 + PDPN+, the cells of these media in the CD31 + CD34- gate could demonstrate lymphatic endothelial-type cells. On the other hand, Gafaar et al. [57] compared endothelial cells derived from umbilical cord blood and differentiated endothelial cells from human adipose mesenchymal stem cells. They reported that the cell from the umbilical cord blood are CD31 + CD144 + CD146 + CD34 + CD73 + CD105 + CD90-CD45- whereas the differentiated endothelial cells are CD90 + CD73 + CD105 + CD34 + CD31+. Therefore, it seems that the CD31 + CD34- gate are differentiated endothelial cells which are positive for CD90, CD73, CD105, and CD31. As D0 had a negligible cells in CD31 + CD34-gate (2.84 ± 2.82%) this theory that these cells in other media’s CD31 + CD34- gate could be differentiated ones may be reinforced.

### Light microscopy analysis of cells in different media

**Fig. 9:**
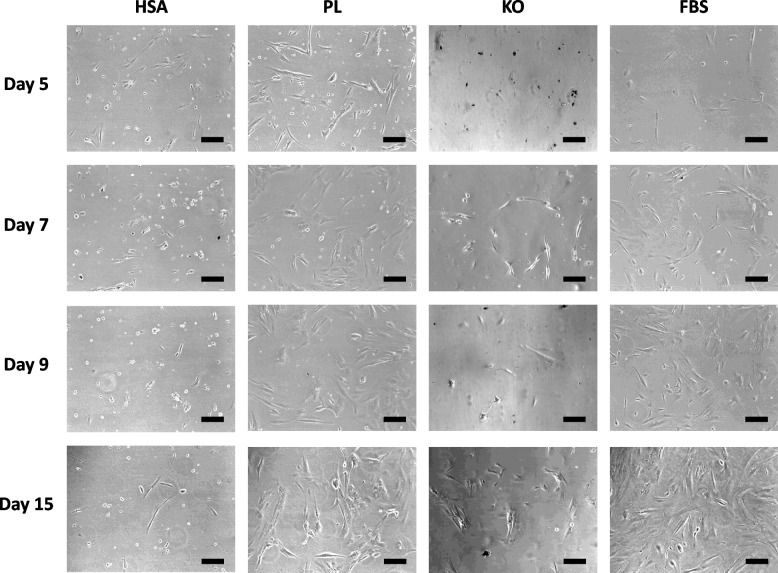
Representative pictures for evaluation of the morphology and population of cells in different media on days 5, 7, 9, and 15 in vitro culture. Scale bars indicate 200 μm

Images of the cells cultured in different media were taken during in vitro culture on days 5, 7, 9, and 15 by using a light microscope (Fig. 9). The microscopy analysis of cells indicated that while the seeding density was similar in all groups at day 0, the population of the cells revealed slight differences among the groups after 3 days of in vitro culture. Cells in PL showed the highest confluence, and those in KO, the lowest. However, when comparing cell growth from day 3 to day 5, 9, and 15, both PL and FBS groups displayed a marked increase in cell number over time with 100% confluence at day 15, while the cell number in the KO and HSA groups showed no notable increase in cells from day 5 to day 15. These differences between the various conditions indicate the potential presence of different cell types when culturing stromal cells by using various supplements.

The cells displayed high adhesion to the flask surface when media supplemented with FBS and PL, and low adhesion when media supplemented with HSA and KO. Cells within all four groups grew in a monolayer and had an elongated, bi- or multipolar cell type, similar to fibroblasts. Within this monolayer, various sizes of cells can be seen, especially in the PL, FBS, and KO-supplemented media. A difference in the HSA group relative to the other groups is the round cells, similar to lymphoblasts. These dissimilarities within the same condition indicate the possibility of more than one cell type in each supplemented medium (Fig. 9).

## Conclusion

In conclusion, our findings show that isolated cells from the ovarian cortex have distinct characterization when cultured in media supplemented with HSA, PL, KO, or FBS. Furthermore, the cells were identified by different phenotypes when they were cultured in media using one of these four additives. Most cells in KO and FBS expressed CD31-CD34-CD73 + CD90+, indicating the possibility of cells being fibroblasts, mesenchymal stem cells, or pericytes in these media. Also, the cells in KO medium indicated a significant increase in cells expressed CD326+ (epithelial cells). PL and HSA groups contained more CD31 + CD34 + CD90 + PDPN+, lymphatic cells compared to the other group as well as PL demonstrated a significant increase in the CD31 + CD34-, endothelial cells compared to day 0.

While this initial study offers a novel strategy to obtain different cell populations for the bioengineered ovary, further studies, such as single-cell RNA-sequencing, would be instrumental in precisely identifying the different cell populations before and after in vitro culture. Identifying cells before their use for regenerative medicine is crucial as different cell types secret different growth factors and cytokines that can direct the fate of engineered tissue and the formation of extracellular matrix.

## Data Availability

Contact the authors for data requests.

## Abbreviations

*DMEM/F12*: Dulbecco’s modified Eagle’s medium F-12 nutrient mixture
*DPBS*: Dulbecco’s phosphate-buffered saline
*FBS*: Fetal bovine serum
*HSA*: Human serum albumin
*KO*: KnockOut serum replacement
*PL*: Platelet lysate
